# Methylation Linear Discriminant Analysis (MLDA) for identifying differentially methylated CpG islands

**DOI:** 10.1186/1471-2105-9-337

**Published:** 2008-08-08

**Authors:** Wei Dai, Jens M Teodoridis, Janet Graham, Constanze Zeller, Tim HM Huang, Pearlly Yan, J Keith Vass, Robert Brown, Jim Paul

**Affiliations:** 1 Ovarian Cancer Action Centre and Section of Epigenetics, Department of Oncology, Imperial College, Hammersmith Hospital, London, UK; 2 Centre for Integrative Cancer Biology, Ohio State University, Columbus, USA; 3 Translational Medicine Research Centre, University of Dundee, UK; 4 The Beatson West of Scotland Cancer Centre, Cancer Research UK Clinical Trial Unit, Glasgow, UK

## Abstract

**Background:**

Hypermethylation of promoter CpG islands is strongly correlated to transcriptional gene silencing and epigenetic maintenance of the silenced state. As well as its role in tumor development, CpG island methylation contributes to the acquisition of resistance to chemotherapy. Differential Methylation Hybridisation (DMH) is one technique used for genome-wide DNA methylation analysis. The study of such microarray data sets should ideally account for the specific biological features of DNA methylation and the non-symmetrical distribution of the ratios of unmethylated and methylated sequences hybridised on the array. We have therefore developed a novel algorithm tailored to this type of data, Methylation Linear Discriminant Analysis (MLDA).

**Results:**

MLDA was programmed in R (version 2.7.0) and the package is available at CRAN [1]. This approach utilizes linear regression models of non-normalised hybridisation data to define methylation status. Log-transformed signal intensities of unmethylated controls on the microarray are used as a reference. The signal intensities of DNA samples digested with methylation sensitive restriction enzymes and mock digested are then transformed to the likelihood of a locus being methylated using this reference. We tested the ability of MLDA to identify loci differentially methylated as analysed by DMH between cisplatin sensitive and resistant ovarian cancer cell lines. MLDA identified 115 differentially methylated loci and 23 out of 26 of these loci have been independently validated by Methylation Specific PCR and/or bisulphite pyrosequencing.

**Conclusion:**

MLDA has advantages for analyzing methylation data from CpG island microarrays, since there is a clear rational for the definition of methylation status, it uses DMH data without between-group normalisation and is less influenced by cross-hybridisation of loci. The MLDA algorithm successfully identified differentially methylated loci between two classes of samples analysed by DMH using CpG island microarrays.

## Background

DNA methylation frequently occurs in mammalian DNA at the 5 position of cytosine in CpG dinucleotides. It has been estimated that over 70% of cytosines of CpG dinucleotides are methylated in the human genome. CpG dinucleotides are under-represented in the genome and methylated CpG dinucleotides predominantly occur within repetitive elements [2]. However, there are CpG rich regions of the genome which generally remain unmethylated [3]. These CpG rich regions are known as CpG islands and are frequently located in the promoter or the first exon regions of approximately 60% of all genes [4]. The unmethylated status of CpG islands is thought to be a prerequisite state to maintain the linked gene in an active transcribed and transcriptional permissive state.

Differential Methylation Hybridisation (DMH) is one of several techniques for examining CpG island methylation at a genome-wide scale that has been applied to the identification of aberrantly methylated gene promoters in various cancers [5-12]. Nouzova *et al*[13] modified the original method by using digestion with a methylation-dependent enzyme, M*cr*BC. This enzyme only cleaves methylated CpG DNA sequences. Within-sample comparison is applied after competitive hybridisation with M*cr*BC digested DNA and undigested (mock digested) DNA labelled with Cy3 and Cy5. If a locus is unmethylated the signal intensities of Cy3 and Cy5 are equivalent, while if methylated the Cy5/Cy3 (undigested/digested) ratio is greater than one. However, no common reference is generally used in the modified DMH method, and the unequal representation of methylated and unmethylated sequences due to competitive hybridisation may reduce sensitivity and specificity to detect differential methylation.

**Figure 1:**
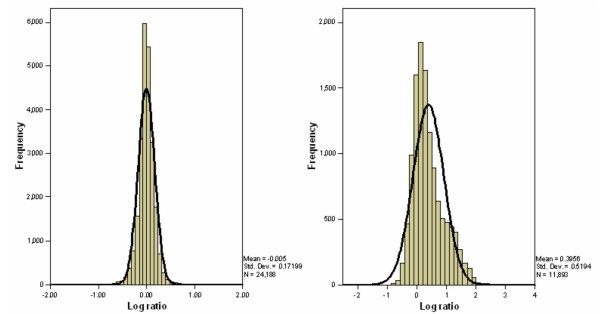
**Distribution of log-transformed ratio of gene expression data in breast cancer and DMH data in A2780 cell line**. The left histogram shows the distribution of log-transformed ratios (cy3/cy5) in gene expression profiling data from a previous study of breast cancer 36] which is symmetric, while the right histogram shows the log-transformed ratios (undigested/digested) of DMH data from the present study which is skewed.

Currently, Significance Analysis of Microarrays (SAM) [14] and Prediction Analysis for Microarrays (PAM) [15] are commonly applied in DNA methylation analysis. Based on the change in hybridisation relative to the standard deviation of repeated measurements, SAM assigns each gene a score that is an extension of the t-statistic. For significant genes with a score over a certain threshold, SAM uses permutations to estimate the false discovery rate (FDR). It has been implemented in many studies of gene expression data [16-21] as well as DMH data, e.g. Wei *et al*. [22] applied SAM to find the differential methylation of CpG island loci between ovarian caner patient groups with short and long progression-free survival (PFS). However, SAM assumes that the microarray data conform to approximate normality and symmetry, leading to the loss of power in the analysis of DMH data that are inherently skewed due to the biological features of DNA methylation in cancer and competitive hybridisation on DMH arrays (Figure 1).

In the modified DMH method, the ratios of raw signal intensities (undigested/digested) greater than 1 reflect the various methylation levels [13]. A ratio cut-off is generally used to identify the hypermethylated loci [7]. However, this is an arbitrary value and does not necessarily accurately reflect the various sources of variation in the experiment. It is therefore desirable to develop an algorithm to more objectively assess the methylation status of loci from DMH data.

PAM is a nearest centroid shrinkage method that identifies those genes that discriminate best between classes. This technique shrinks the class gene centroid towards the overall centroid by a "threshold" amount after standardizing each gene by its within class standard deviation. The "threshold" is identified by cross-validation. This approach was applied in the study by Wei *et al*. [22] and showed certain power in the identification of differentially methylated loci, but PAM is designed for class prediction rather than class comparison. Although the class predictor used in PAM can reflect the difference between classes, a large number of loci actually differentially methylated between the classes are excluded to improve the accuracy of prediction.

Although normalisation has become a standard procedure for the study of microarray data and is necessary for SAM and PAM analysis, unbalanced shifts in methylation status between class samples in DMH limit the use of between-class normalisation which assumes the changes are roughly symmetric. Thus, the differential methylation can be masked by the over-correction of normalisation and it would be preferable to use a method of analysis that does not require normalisation of the data.

Since PAM and SAM may have limitations for analysing DMH data, we have developed an alternative approach based on the specific features and known biological properties of the arrays used for DMH analysis. The algorithm is named as Methylation Linear Discriminant Analysis (MLDA) and has been applied to identify a set of loci differentially methylated between ovarian cisplatin sensitive and resistant cancer cell lines.

## Results

### Outline of MLDA

**Figure 2:**
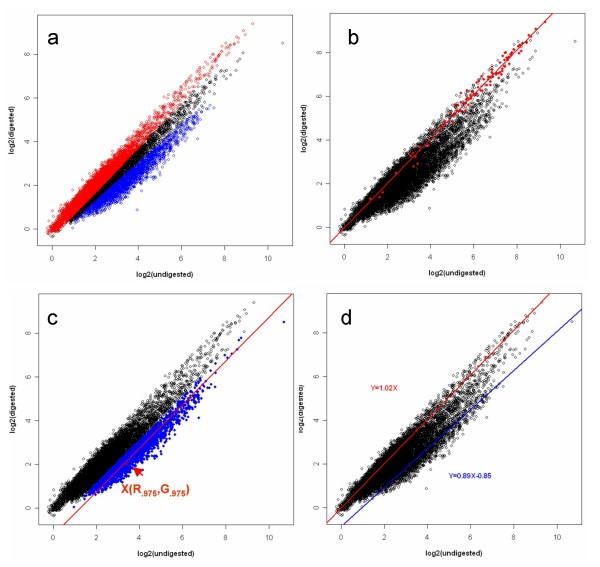
**An illustration of unmethylated and methylated model construction in MLDA in A2780 cell line**. a: Three patterns can be observed on the scatter plot of log-transformed Cy3 (undigested) against log-transformed Cy5 (digested) intensities. b: The unmethylated model constructed using 94 mitochondrial sequences as a unmethylation reference. c: The intermediate model constructed through the 97.5 quantile residual. The point X is the 97.5 quantile residual. The microarray probes colored in blue (standardised residual to the intermediate model is less than 2) are selected to construct the methylated model. d: Methylated (in blue) and unmethylated (in red) models in A2780 cell line.

In this study, we have developed a novel approach, named MLDA, for analysing CpG island microarray hybridisation data that allows the identification of differentially methylated loci. MLDA was programmed in R (version 2.7.0) and the package is available at CRAN [1]. This approach uses three relatively simple linear regression models. The first one is constructed by the log-transformed signal intensities of unmethylated features and used as the reference for unmethylation (Figure 2b). The second one is the intermediate model constructed through the point corresponding to the 97.5-quantiles residual below the first linear regression line (Figure 2c). The features with a standardised residual less than 2 from this intermediate model are used to generate the third model which is used as the reference for methylation (Figure 2d). The log likelihood ratio of a locus being methylated is then proportional to the difference between the squared standardised residual from the methylated line and that from the unmethylated line. The log likelihood threshold of zero then provides a more rational basis for distinguishing between methylated and unmethylated loci than a robust undigested/digested ratio of 1.5, as it takes into account the observed variability in the experiment.

**Figure 3:**
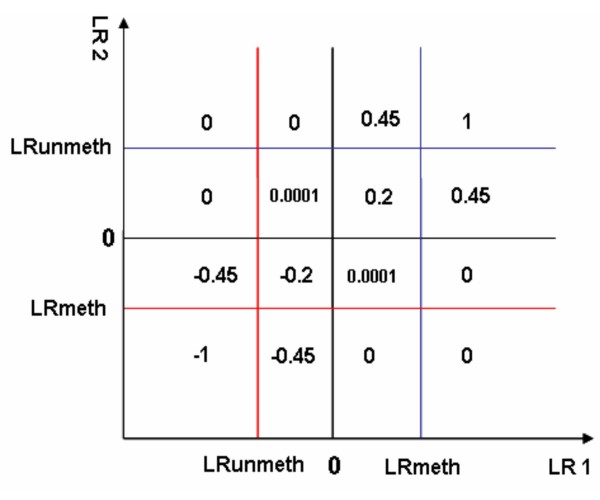
**Weighted scoring scheme**. The microarray probes consistently identified as methylated candidates on dye-swap arrays were scored 1; similarly unmethylated microarray probes were scored -1. The rest of the microarray probes were assigned a weighted score based on their location on the plot. LRmeth: log likelihood ratio cut-off for methylated loci; LRunmeth: log likelihood ratio cut-off for unmethylated loci. LR: log likelihood ratio on dye-swapped arrays.

In our approach the consistency and inconsistency rates of log likelihood ratios on dye-swapped/duplicate arrays are used to determine methylation and unmethylation cut-offs, which keep the consistency rate (CR) relatively high (about 140%) and the inconsistency rate (IR) low (about 1%). Each loci is assigned a score based on the cut-offs using the weighted methylation scoring scheme. The feature consistently identified as methylated candidates on dye-swapped/duplicate arrays are scored as 1; similarly unmethylated features are scored as -1; the rest of the feature are assigned a weighted score corresponding to their location on the plot of log-likelihood ratios (Figure 3).

The averaged score for each locus is calculated in each sample class (e.g. resistant or sensitive) and plotted against each other. A robust regression model is then fitted to these data. The standardised residuals from the robust regression model are assumed to follow a normal distribution *N*(*μ*, *σ*^2^). The outliers of the standardised residuals are identified as the differentially methylated loci between the class samples.

### DMH dataset

**Figure 4:**
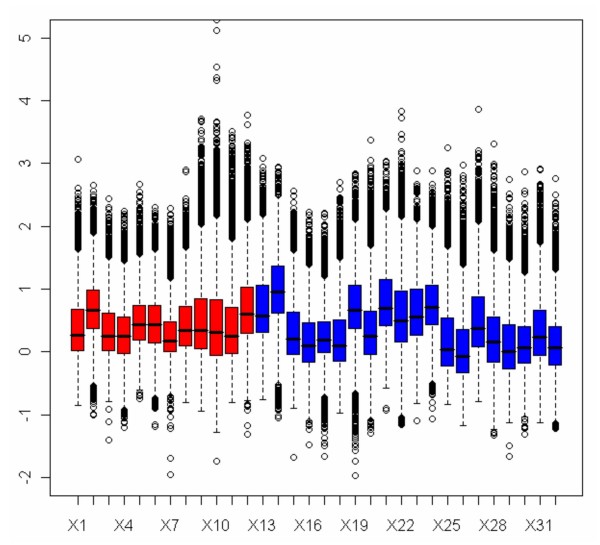
**Box plot of log ratios of undigested signal intensities against digested signal intensities in 16 cell lines (dye-swapped arrays)**. The boxes colored in red are the A2780 sensitive cell lines; in blue are the A2780 resistant cell lines. As normalisation is not applied, the center and scale of log ratios for the 16 cell lines are not at the same level.

MLDA was applied to identify the CGIs differentially methylated from DMH data derived from sensitive A2780 derivatives (A2780, A2780p3, A2780p5, A2780p6, A2780p13, A2780p14) and isogenically matched, resistant lines [23] derived by multiple exposures to cytotoxic levels of cisplatin and which are 2–5 fold resistant to cisplatin in clonogenic assays (A2780cp70, A2780/MCP1, A2780/MCP2, A2780/MCP3, A2780/MCP4, A2780/MCP5, A2780/MCP6, A2780/MCP7, A2780/MCP8, A2780/MCP9). After background correction, the log-transformed digested and undigested intensities of the 13056 microarray probes show three approximately parallel linear patterns (Figure 2a). The first pattern (digested/undigested is close to 1) represents the unmethylated sequences. The second pattern represents either hemi-methylated sequences or the unmethylated sequences cross-hybridised with the methylated ones on the panel. The third pattern represents the methylated sequences in target DNA. The methylated and unmethylated loci in target DNA can be characterised by a linear regression model for each pattern. As previously mentioned, normalisation may not be appropriate for DMH data, so the log ratios of signal intensities in two classes of samples are not at the same level (Figure 4). Normalisation is not required for MLDA as the determination of the methylation score is based on the data within each experiment.

Mitochondrial DNA is unmethylated [24], therefore, the signal intensities of both channels of microarray probes for mitochondrial sequences are expected to be equal. However, a bi-modal distribution is observed in the log-transformed fluorescence ratios (digested/undigested) of 121 mitochondrial sequences. The first peak represents the unmethylated mitochondrial sequences and the second lower peak is assumed to be the mitochondrial sequences cross-hybridised with other methylated sequences on the panel. Thus, we selected 94 of 121 mitochondrial sequences that were consistently unmethylated through all the cell lines and used them as the unmethylation reference in target DNA.

**Figure 5:**
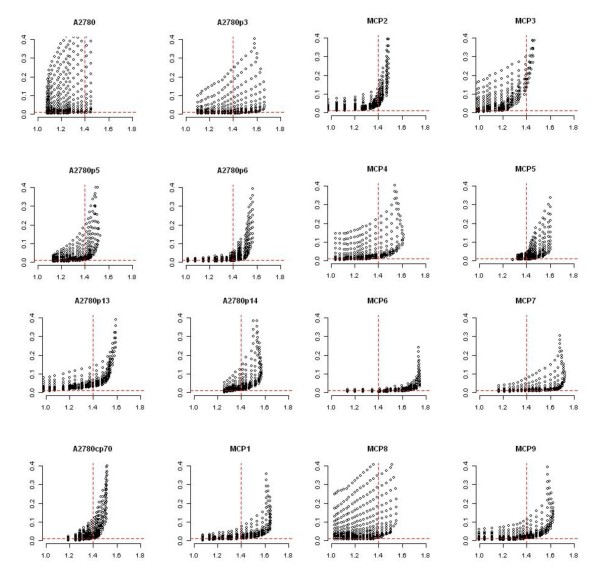
**CR against IR in 16 cell lines**. X axis is the consistency rate (CR) and y axis is the inconsistency rate (IR). IR tends to rise with the increase of CR slowly, but starts to increase dramatically when the CR goes above 140%, at which point the inconsistency rate is generally about 1%. Not all cell lines could reach this point e.g. MCP3.

**Figure 6:**
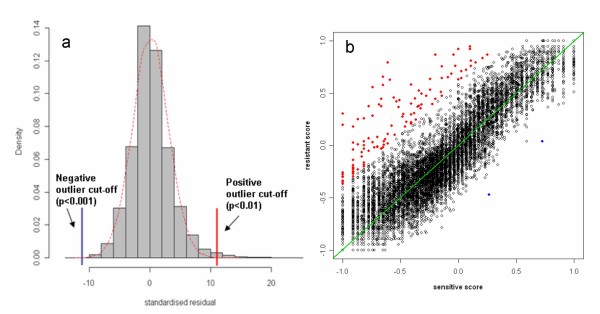
**Outliers identifications**. a: Distribution of the observed (histogram) standardised residuals and the theoretical distribution based on the fitted model (dashed smooth line in red). The red and blue solid line are the positive and negative cut-offs, respectively. b: Scatter plot of sensitive scores against resistant scores in A2780 series cell lines. The hypermethylated loci are colored in red and hypomethylated loci are in blue. The robust regression model is Y = 0.9956X + 0.0019.

The parameters of those two models in all 16 cell lines were estimated (Table 1). The slope of the unmethylated regression line constructed by 94 mitochondrial sequences is indeed close to 1. After computing the log-likelihood ratios, the methylation and unmethylation cut-offs and associated IRs and CRs were determined from the dye-swapped array pairs (details in Method section). As shown in Figure 5, IR tends to rise with the increase of CR slowly, but starts to increase dramatically when the CR goes above 140%, at which point IR is generally about 1%. We have therefore used CR > 140% and IR < 1% as the criteria for determining the methylation and unmethylation cut-offs. Each locus was scored using the weighted scoring scheme based on those cut-offs. The averaged scores in 6 cisplatin-sensitive cell lines and 10 cisplatin-resistant cell lines were used to construct a robust regression model. Figure 6a shows that the standardised residuals (residual/*σ*) from the robust regression model roughly follow a normal distribution. The positive and negative outliers are determined as described in Method section.

**Table 1:** Parameters of linear models in MLDA for 16 cell lines in DMH dataset I

**Unmethylation linear regression model**								
cell line	intercetp (*α*)	slope (*β*)	*σ*	R2	intercept_ds _(*α*)	slope_ds _(*β*)	*σ*_ds_	R2_ds_
A2780	-0.0003	1.0122	0.1727	0.9829	0.0005	1.0574	0.1897	0.978
A2780p3	-0.0003	1.0343	0.1212	0.9897	0.0018	1.1065	0.1425	0.9882
A2780p5	-0.0003	1.0138	0.1684	0.984	-0.0002	1.0728	0.1425	0.9883
A2780p6	0.0002	0.9914	0.1605	0.9778	-0.0001	1.0012	0.1638	0.9747
A2780p13	-0.0012	1.024	0.1628	0.9786	-0.0005	1.0744	0.1436	0.9852
A2780p14	-0.0022	1.0499	0.1523	0.9809	-0.0009	1.034	0.2069	0.9691
A2780cp70	0.0013	0.9604	0.2532	0.9524	-0.0002	1.0119	0.2402	0.9479
MCP1	0.0002	0.9946	0.145	0.9803	-0.0023	1.112	0.1452	0.9836
MCP2	0.0000	0.998	0.137	0.9727	-0.0028	1.0475	0.1719	0.9653
MCP3	0.0004	0.9932	0.2253	0.9183	-0.0023	1.0517	0.2795	0.8978
MCP4	0.0006	0.9838	0.1739	0.9718	-0.0028	1.077	0.1947	0.9751
MCP5	0.0009	0.9857	0.2464	0.9639	-0.0008	1.017	0.2166	0.9692
MCP6	-0.0022	1.0352	0.122	0.9751	-0.0068	1.1283	0.154	0.9752
MCP7	-0.0005	1.0079	0.1379	0.9791	-0.0045	1.1529	0.1588	0.9764
MCP8	-0.0028	1.0578	0.1903	0.9431	-0.0068	1.1193	0.1885	0.9575
MCP9	-0.0017	1.0331	0.1834	0.9614	-0.0091	1.1538	0.1691	0.9674

**Methylation linear regression model**								
cell line	intercetp (*α*)	slope (*β*)	*σ*	R2	intercept_ds _(*α*)	slope_ds _(*β*)	*σ*_ds_	R2_ds_

A2780	-0.8839	0.8917	0.1491	0.9438	-0.8055	0.9086	0.1706	0.926
A2780p3	-1.1672	0.9797	0.1518	0.9553	-0.7414	0.9774	0.179	0.9438
A2780p5	-0.8991	0.8978	0.1515	0.9476	-0.9246	0.9766	0.1673	0.9523
A2780p6	-0.9455	0.9562	0.1838	0.9378	-1.1995	0.9641	0.1788	0.9324
A2780p13	-1.8918	0.9807	0.2535	0.8962	-1.8049	0.9652	0.2936	0.8512
A2780p14	-1.5637	0.9142	0.2549	0.8857	-1.4468	0.9066	0.2128	0.8837
A2780cp70	-1.0317	0.8501	0.1581	0.9115	-1.3074	0.8967	0.1541	0.9265
MCP1	-1.199	0.9781	0.1692	0.9467	-1.0935	1.0384	0.1775	0.9525
MCP2	-0.8037	0.9292	0.1486	0.9557	-0.9738	0.9381	0.2176	0.8848
MCP3	-1.1244	0.9151	0.1755	0.9482	-0.9303	0.9205	0.2599	0.8098
MCP4	-1.4326	0.9171	0.1418	0.966	-1.6205	0.961	0.2348	0.8323
MCP5	-1.1187	0.9425	0.1839	0.9404	-1.2007	0.9546	0.1757	0.9295
MCP6	-1.246	0.925	0.1966	0.9294	-1.2182	0.9826	0.2248	0.8989
MCP7	-1.8972	0.9909	0.1977	0.9442	-1.4894	1.0139	0.2458	0.8886
MCP8	-1.0219	0.9905	0.1975	0.9468	-0.4735	0.9421	0.228	0.8761
MCP9	-1.3399	0.9837	0.2073	0.9352	-1.1497	1.0078	0.1967	0.9115

ds: dye swap

*σ*: standard deviation

R^2^: coefficient of determination

Finally, 115 loci were identified as candidates differentially methylated between A2780 sensitive and these resistant cell lines (additional file 1). Noticeably, 113 of 115 loci (*p *= 8.8 × 10^-3^, outlier detection test [25]) were hypermethylated, but only 2 loci (*p *< 0.001, outlier detection test) lost methylation in the resistant cell lines (Figure 6b). This is consistent with the unbalanced shift in DMH data and indicates cisplatin treatment of cells selects preferentially for hypermethylation of loci, rather than hypomethylation in these tumor cells.

### Validation of differential methylation

To confirm the differential methylation of loci identified in this study, we experimentally tested the methylation of 26 loci by methylation-specific PCR (MSP) and/or pyrosequencing of bisulphite modified DNA [26] in sensitive A2780 derivatives and cisplatin resistant derivatives. Twenty-three out of the 26 loci have been confirmed as differentially methylated (additional file 1). It should be noted that MSP and pyrosequencing only examine methylation at a limited number of CpG sites of the sequence present on the DMH analysis. It is possible that the loci which were not confirmed as differentially methylated are methylated at other CpG sites which are detected by DMH but not targeted by MSP and/or pyrosequencing primers and so 23 out of 26 loci confirmed as differentially methylated is a minimum estimation.

To compare the results from MLDA, SAM and PAM, we analysed the DMH dataset by all three methods. MLDA identified 115 loci (113 hypermethylated and 2 hypomethylated loci, misclassification error < 0.001), SAM identified 152 loci (149 hypermethylated and 3 hypomethylated loci, misclassification error = 0.227, FDR = 6.17 × 10^-3^), and PAM found 24 hypermethylated loci (misclassification error = 0.084, FDR < 0.001) in the resistant cell lines. Twenty-four loci identified by all three methods are listed in Table 2.

**Table 2:** 24 loci identified by MLDA, PAM and SAM as differentially methylated candidates in the comparison between A2780 cisplatin sensitive and cisplatin multiple-selected resistant cell lines.

**microarray ID**	**status**	**validation**	MLDA rank*	**PAM rank**	**SAM rank**	CGI***	gene symbol**	**GenBank Accession**	**Chromosome**
66_G_6	hypermethylated	Yes	1	1	1	Yes			
121_D_9	hypermethylated	Yes	2	5	6	Yes	CRABP1	NM_004378	15
39_E_1	hypermethylated	ND	3	2	2	No			
122_D_9	hypermethylated	No	4	11	11	Yes	SOX12	NM_006943	20
123_D_9	hypermethylated	No	5	10	10	Yes	SOX12	NM_006943	20
51_H_8	hypermethylated	Yes	6	18	19	No	FEZF2	NM_018008	3
58_A_1	hypermethylated	ND	7	22	22	Yes			
80_H_5	hypermethylated	ND	8	14	16	No			
21_A_11	hypermethylated	Yes	9	17	18	Yes	NTN4	NM_021229	12
38_D_7	hypermethylated	Yes	11	23	24	Yes	AGBL2	NM_024783	11
40_E_1	hypermethylated	ND	12	19	17	No			
18_A_7	hypermethylated	ND	13	20	20	No	EDIL3	NM_005711	5
55_F_8	hypermethylated	Yes	14	9	9	Yes	BC127881	BC127881	7
122_B_1	hypermethylated	ND	15	16	14	No			
109_A_6	hypermethylated	No	18	12	13	Yes			
41_D_9	hypermethylated	Yes	22	8	8	Yes	WNT1	NM_005430	12
42_D_9	hypermethylated	ND	23	4	4	No			
119_A_6	hypermethylated	Yes	24	6	5	Yes	NR2E1	NM_003269	6
63_A_8	hypermethylated	ND	26	15	15	No			
6_D_4	hypermethylated	Yes	31	3	3	Yes	LMX1A	NM_177398	1
17_H_9	hypermethylated	Yes	34	13	12	Yes	HRASLS3	NM_006290	6
5_D_4	hypermethylated	Yes	35	7	7	Yes	LMX1A	NM_177398	1
24_D_3	hypermethylated	Yes	75	24	23	Yes	SP5	NM_001003845	2
122_G_1	hypermethylated	ND	101	21	21	Yes			

ND: not done. Yes: validated. No: not validated

MLDA rank*: the rank of standardised residuals to the robust regression line constructed by the averaged sensitive scores against averaged resistant scores

Gene symbol**: only the gene of which transcription start site (TSS) is within 5 kb span of the loci

CGI***: CpG island defined by Gardiner-Garden and Frommer [31].

## Discussion

Hypermethylation of promoter CpG islands is strongly correlated to transcriptional gene silencing and epigenetic maintenance of the silenced state and is a potential rich source of biomarkers of cancer. Differential Methylation Hybridisation (DMH) is one technique used for genome-wide DNA methylation analysis. The study of such microarray data sets should ideally account for the specific biological features of DNA methylation and the non-symmetrical distribution of the ratios of unmethylated and methylated sequences hybridised on the array. We have therefore developed a novel algorithm tailored to this type of data, Methylation Linear Discriminant Analysis (MLDA). MLDA utilises log likelihood ratios representing the relative probability that loci are methylated instead of log ratios of signal intensities used in previous studies [6-10,27]. Validation of 23/26 identified loci using independent methods of methylation analysis shows that MLDA can robustly identify differential methylated loci between ovarian cancer sensitive and resistant cell lines without requiring the data to be normalised.

Although a log likelihood ratio above zero means that the locus tends to be methylated, we did not use zero as the cut-off to determine the number of methylated and unmethylated sequences, as the existence of cross-hybridisation and measurement errors in the DMH assay makes this unreliable. To increase the precision of the methylation classification, we used the inconsistency (IR) and consistency (CR) rates between the dye-swap arrays to determine likelihood ratio cut-offs for methylation and unmethylation and assigned each locus a methylation score based on the position relative to these cut-offs. As shown in Figure 5, not all cell lines can reach the point that CR is around 140% and IR is about 1%. IR and CR need to be carefully selected as the methylation scores of loci are consequently influenced by the change of IR and CR. We also observed a lower CR (about 120%) and a higher IR (about 2%) in another CpG island array using DMH (data not shown), therefore, further examination of what factors influence the achievable CR and IR rates may improve the utility of the MLDA approach.

Data on methylation status for 121 mitochondrial derived sequences were available in this study. Mitochondrial sequences would be expected to be unmethylated. We used 94 mitochondrial sequences to construct unmethylated linear model at the beginning of the study, and indeed, 93 of 121 mitochondrial loci were defined as unmethylated and 25 loci being of uncertain methylation status by MLDA. However, three mitochondrial loci were identified as hypermethylated candidates in the resistant ovarian carcinoma cell lines by both MLDA and SAM. One explanation of this discrepancy is that all these three loci have more than one BLAT hit indicating the existence of homology with nuclear DNA sequences, raising the possibility of hybridisation with these nuclear DNA sequences which may be differentially methylated. As shown in Figure 2a, the loci in the middle pattern represent either hemi-methylated sequences or the unmethylated sequences cross-hybridised with the methylated ones on the panel. No specific allowance is made for these intermediate points in analysis by SAM and PAM, whereas MLDA attempts specifically to down-weight these points in the identification of the methylation regression line. By giving a lower weighted score (close to 0) (Figure 3) to those loci, MLDA reduces the influence of cross-hybridisation among this group of sequences. Of course cross-hybridisation may also occur in the loci in the other two patterns (methylated and unmethylated patterns), but it is not possible for any mathematical approach to identify this.

The misclassification error of MLDA based on the methylation score is much lower than that for either SAM or PAM based on the log ratios, indicating the potential of MLDA methylation scores to be used as a reliable discriminator between classes of samples.

## Conclusion

We have developed a novel method, named MLDA, for genome-wide DNA methylation studies. MLDA can transform the signal intensities to log-likelihood ratios through three linear regression models. Using this approach MLDA allows determination of the methylation status of a locus based on dye-swapped/duplicate arrays. The method has been applied to assess the methylation status of each locus and identified 115 loci that exhibit differential methylation between A2780 sensitive and resistant cell lines. A minimum of 23 out of 26 loci have been confirmed by independent methods as differentially methylated.

## Methods

First, all intensity values were log transformed. A multiplicative background correction was applied to correct signal intensities for the background noise in each array. After background correction, the log-transformed digested and undigested intensities show three approximately parallel linear patterns (Figure 2a). The first pattern (digested/undigested is close to 1) represents the unmethylated sequences. The second pattern represents either hemi-methylated sequences or the unmethylated sequences cross-hybridised with the methylated ones on the panel. The third pattern represents the methylated sequences in target DNA. The methylated and unmethylated loci in target DNA can be characterised by a linear regression model for each pattern. The distance of each spot to the methylated and unmethylated lines respectively can then be estimated by standardised residuals. The log likelihood ratio of a locus being methylated is then proportional to the difference between the squared standardised residual from the methylated line and that from the unmethylated one. The algorithm based around this regression approach is named Methylation Linear Discriminant Analysis (MLDA) and was programmed in R version 2.7.0.

### Log-likelihood ratio transformation

a An univariate linear regression model was constructed for the unmethylated probes (e.g. mitochondrial derived features) using formula (1) where *α *is the intercept, *β *is the slope of the model, and *ξ *is the error representing the unpredicted or unexplained variation in the model (Figure 2b). The parameters of regression line were estimated by the method of least squares (formula 2 and 3).

(1) *G*_*i *_= *α *+ *βR*_*i *_+ *ξ*_*i *_*i *= 1,2,3.......*k*

(2) $$\begin{array}{cc} \hat{\beta }=\frac{\sum \left(R_{i}-\bar{R})(G_{i}-\bar{G}\right)}{\sum {\left(R_{i}-\bar{R}\right)}^{2}} & i=1,2,3.......k \end{array}$$

(3) $$\hat{\alpha }=\hat{\beta }\bar{R}-\bar{G}$$

*k *is the number of unmethylated controls on DMH array. *G*_*i *_and *R*_*i *_are the logarithmic-transformed digested and undigested intensities of microarray probes for mitochondrial sequences, respectively. $\bar{G}$ and $\bar{R}$ are the averaged logarithmic-transformed undigested and digested intensities of the k unmethylated controls.

b. The scale estimate *σ*_mito _associated with the error term in the linear regression model was estimated from the residuals from the observed *k *points to the fitted line. The most extreme 10% of residuals was omitted from either end of the distribution to minimise the impact of extreme residuals on this estimate.

c. The standardised residuals of all the microarray probes to the unmethylation regression line were calculated as formula (4).

(4) $$SR_{mito}=\frac{residuals_{mito}}{\sigma_{mito}}$$

d. The point corresponding to the 97.5-quantiles residual below the unmethylation line is represented as X (R.975, G.975). The intermediate linear model (Figure 2c) was constructed through point X with a slope assumed to be 1 and the intercept estimated as formula (5).

(5) $$\hat{\alpha }=G_{.975}-R_{.975}+1.96\sigma_{mito}$$

e. The standardised residuals of all the microarray probes to the line with slope 1 and intercept estimated from (5) were calculated as formula (6). The variance of the residuals to the intermediate model was assumed to be similar as that in the mitochondrial model.

(6) $$SR_{.975}=\frac{residuals_{.975}}{\sigma_{mito}}$$

f. The microarray probes with standardised residuals less than 2 were included for later robust regression analysis. The line estimated from this regression analysis represents the methylation regression line (Figure 2d).

g. The scale estimate *σ*_meth _of the methylation regression line was estimated using only those microarray probes below the line, with the most extreme 5% removed.

h. The standardised residuals of all the microarray probes to the methylated regression line were calculated as formula (7). The log likelihood ratio (LR) of all the microarray probes was estimated by formula (8) for further analysis.

(7) $$SR_{meth}=\frac{residuals_{meth}}{\sigma_{meth}}$$

(8) *LR *= *SR*^2^_*mito *_- *SR*^2^_*meth*_

### Determination of log likelihood ratio cut-offs

**Figure 7:**
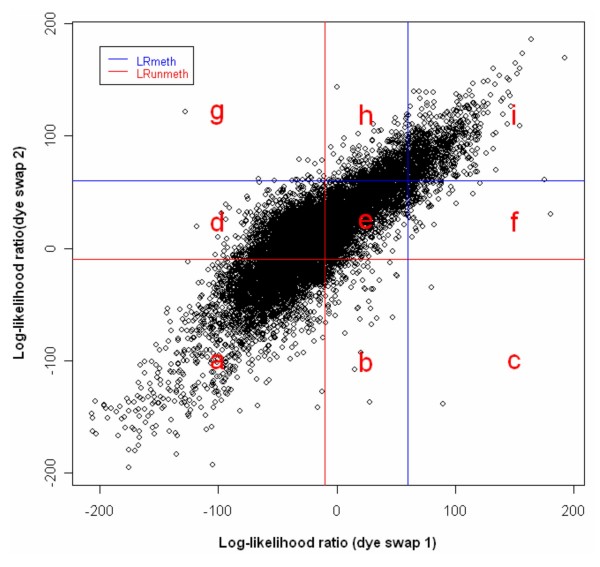
**Determination of methylation and unmethylation cut-offs of likelihood ratios on dye-swapped arrays**. LRmeth: log likelihood ratio cut-off for methylated spots; LRunmeth: log likelihood ratio cut-off for unmethylated spots.

Two inconsistency rates (IR_meth _and IR_unmeth_) and two consistency rates (CR_meth _and CR_unmeth_) between dye-swap arrays were used to determine the log like likelihood ratio threshold. IR_meth _(formula 9) represents the rate of the microarray probes identified as methylated in one array but as unmethylated in the other one, while IR_unmeth _(formula 10) is the rate of the microarray probes identified as unmethylated in one array but as methylated in the other one. CR_meth _(formula 11) and CR_unmeth _(formula 12) are the rates for the spots identified as methylated (CR_meth_) and unmethylated (CR_unmeth_) in both dye-swap arrays (Figure 7).

**Table 3:** Classification of loci

	Unmethylated	uncertain	methylated
Unmethylated	a	b	c
Uncertain	d	e	f
Methylated	g	h	i

(9) $$IR_{meth}=\frac{c}{c+f+i}+\frac{g}{g+h+i}$$

(10) $$IR_{un\;meth}=\frac{g}{a+d+g}+\frac{c}{a+b+c}$$

(11) $$CR_{meth}=\frac{i}{c+f+i}+\frac{i}{g+h+i}$$

(12) $$CR_{meth}=\frac{a}{a+b+c}+\frac{a}{a+d+g}$$

b The log likelihood ratio thresholds (LR_meth _and LR_unmeth_) for methylated and unmethylated microarray probes, which kept the IR rates low (at or close to 1%) and the CR rates high (at or close to 140%), were used as the cut-offs for methylated and unmethylated loci. IR tends to rise with the increase of CR slowly, but starts to increase dramatically when the CR goes above 140%, at which point the inconsistency rate is generally about 1%. We have therefore used CR > 140% and IR < 1% as the criteria for determining the methylation cut-offs.

### Identification of robust regression outliers

Each microarray probe was scored based on the cut-offs of likelihood ratios for methylation and unmethylation on dye-swap arrays using the weighted methylation scoring scheme shown in Figure 3. The microarray probes consistently identified as methylated candidates on dye-swap arrays were scored of 1; similarly unmethylated microarray probes were scored of -1. The rest of the microarray probes were assigned a weighted score based on their location on the plot.

A robust regression model [28] was constructed with the averaged scores in one class of samples as the explanatory variable, and the corresponding scores in the other class of samples as the dependent variable. The degree of trimming was determined according to Barnett *et al*. [29] when estimating the variance of residuals to the robust linear regression model.

It was assumed that the standardised residuals (SRs) from the robust regression line followed a normal distribution *N*(*μ*, *σ*^2^). *μ *and *σ *were estimated excluding outliers using the MAD-Median Rule [30]. The p value for each SR cut-off was calculated as described by Simon *et al *[25]. This p-value reflects the probability of observing a group of more extreme residuals from the fitted normal distribution. Microarray probes were identified as outliers if their SRs were larger than the cut-off for which the p-value was less than 0.01.

### Estimation of misclassification rate

The misclassification rate was estimated by drawing bootstrap samples 500 times with replacement from the two classes (sensitive and resistant) and carrying out hierarchical clustering based on the loci identified as differentially methylated using weighted scores for MLDA and log ratios without between-group normalisation for SAM and PAM, respectively. Clustering was carried out using Euclidean distance as the distance metric, and clusters were agglomerated using the average linkage criterion. The clustering tree was cut into two groups and the number of misclassified cell lines was counted. The misclassification rate was obtained from the averaged number of misclassified samples in 500 bootstraps divided by the total number of samples.

### SAM and PAM analysis

The raw signal intensities of each channel were subtracted by the median signal intensities of corresponding channel of controls on HCGI12K array. After this correction, SAM in samr package and PAM in pamr package were applied using log ratios (digested/undigested) in R version 2.7.0. Between-group normalisation was not used in SAM and PAM to avoid over-correction masking the differential methylation.

## Authors' contributions

WD conducted the statistical analysis and algorithm development supervised by JP and RB. The DMH data was produced by JMT in collaboration with TH and PY. RB, JP and KV conceived the study. JMT, JG and CZ conducted validation by MSP or pyrosequencing in RB's lab. WD, JP and RB prepared the manuscript with review by all authors. Funding was obtained by RB and TH.

## Supplementary Material

Additional file 1 115 differential methylated candidates identified by MLDA in A2780 series cell lines. Click here for file
